# Perceptions on Diet and Dietary Modifications during Postpartum Period Aiming at Attenuating Progression of GDM to DM: A Qualitative Study of Mothers and Health Care Workers

**DOI:** 10.1155/2018/6459364

**Published:** 2018-08-26

**Authors:** Thamudi D. Sundarapperuma, Champa J. Wijesinghe, Priyadarshika Hettiarachchi, Sudharshani Wasalathanthri

**Affiliations:** ^1^Department of Nursing, University of Ruhuna, Galle 80000, Sri Lanka; ^2^Department of Community Medicine, University of Ruhuna, Galle 80000, Sri Lanka; ^3^Department of Physiology, University of Sri Jayewardenepura, Nugegoda 11222, Sri Lanka; ^4^Department of Physiology, University of Colombo, Colombo 00800, Sri Lanka

## Abstract

**Background:**

Gestational diabetes mellitus (GDM) is a global concern. GDM mothers have a 7-fold relative risk of developing type 2 diabetes mellitus (T2DM) in their later life. User-friendly and culturally acceptable dietary interventions can minimize this risk. Therefore, this study aims at exploring the perceptions of GDM mothers and health care workers regarding factors that influence postpartum dietary practices aimed at attenuating the trajectory from GDM to DM.

**Methods:**

The study was conducted in selected MOH areas in three districts of Sri Lanka. Six focus group discussions were conducted with thirty mothers with a history of GDM and six in-depth interviews with six health care workers. The phenomenon of interest was to obtain inputs of two stakeholder groups on healthy food habits of GDM mothers during the postpartum period. Framework analysis was used to analyse the data. Data were coded using the analytical framework, abstracted from transcripts, and summarized verbatim in Microsoft Excel in a matrix comprised of one row per participant and one column per code. Finally, the matrix was reviewed intensely and themes were generated.

**Results:**

Overall, seven themes emerged from both cases: (1) myths and traditions specific to the postpartum period, (2) lack of motivation, (3) time pressure, (4) financial barriers, (5) negligence of mothers and families, (6) lack of awareness regarding GDM and its postpartum dietary recommendations, and (7) cultural barriers.

**Conclusions:**

This study provides an insight into the existing knowledge, common practices, and attitudes regarding food habits among postpartum mothers with a history of GDM. Since the postpartum period is unique, identifying barriers is crucial when introducing dietary modification protocols in order to prevent or attenuate the progression of GDM to T2DM in these mothers. The knowledge gained will be used to introduce feasible, scientifically sound, and culturally acceptable postpartum dietary recommendations for GDM mothers.

## 1. Introduction

Gestational diabetes mellitus (GDM) is a state of glucose intolerance commencing with the onset of pregnancy, or first recognized during pregnancy [1]. Due to the current economic, social, and nutritional transition, an increasing prevalence of GDM is noted globally [2]. Women with a previous history of GDM have a high risk of recurrence of the same in subsequent pregnancies [3] and also has a 7.43-fold relative risk of developing type 2 diabetes mellitus (T2DM) compared to women without GDM [4]. Although ethnicity, prepregnancy weight, age, family history of diabetes, and glycaemic indices during pregnancy are nonmodifiable risk factors favouring progression of GDM to overt diabetes [5], increase in BMI is a risk factor [6] modifiable by appropriate intervention.

Life style interventions attenuate the progression of GDM to T2DM [4, 7] delaying or preventing the development by one-sixth times [8]. Weight reduction by increased physical activity and limiting the consumption of calories reduces the risk of diabetes [9]. Previous studies have suggested reducing the postpartum energy intake by limiting the consumption of energy-dense foods such as fast foods, high-fat snacks, fried foods and sugar-sweetened beverages, reducing the portion size, increasing fruit and vegetable intake, and modifying ethnic recipes accordingly [10–12]. Carolan et al. [13] recommended that these interventions should be culturally acceptable to get the optimal benefit.

Postpartum mothers are a special group who face difficulties in maintaining healthy eating patterns and regular physical activities [14]. Therefore, a better understanding of the barriers to behaviour changes is needed when planning out lifestyle interventions. Many factors such as extreme tiredness, time and financial constraints [15], needs of the newborn, and demands of the family [16] act as obstacles for healthy lifestyles. In addition, food preferences of other family members and confidence and skills in cooking healthy food were other psychosocial factors influencing postpartum dietary behaviours [17]. A careful exploration and analysis of these factors is therefore important when designing nutrition interventions for postpartum mothers.

Though several studies have been done to determine the perception of mothers [17–19] and health care workers [20, 21] regarding diet during the postpartum period, there is a dearth of evidence emerging from South Asian cultures. Since postpartum mothers are faced with unique issues, findings of studies on dietary perceptions of diabetic patients [22] are not directly applicable to them. Cultural beliefs, traditions, and social influences can directly affect the food habits of postpartum mothers, and these should be taken into account when implementing dietary guidelines to reduce the risk of diabetes [7, 14, 23]. Therefore, the aim of this study was to explore the perceptions of GDM mothers and healthcare workers regarding factors that influence postpartum dietary practices, for the purpose of attenuating the trajectory from GDM to T2DM.

## 2. Materials and Methods

### 2.1. Study Design and Settings

This study was a community-based, phenomenological qualitative study carried out in three selected districts in Sri Lanka, namely, Colombo, Gampaha, and Galle.

Focus group discussions (FGDs) were used to explore the perceptions of mothers regarding postpartum dietary practices in order to allow cross talk between them and to generate ideas freely. Thirty volunteering antenatal mothers with a history of GDM, who spoke the native language, Sinhala, who were not employees of the health sector were recruited. Five to ten mothers were invited for each FGD, as smaller groups are more manageable to obtain optimum results [24].

In-depth interviews (IDIs) were chosen to assess the perspective of healthcare workers as IDIs allow detailed exploration of the reactions of respondents without contamination [25]. Further, IDIs ensured longer speaking time and privacy during information gathering which could be challenging to the health care workers. Three public health midwives and three postnatal nurses were recruited as they were believed to have the closest contact with mothers. All healthcare workers were required to have at least 3 years of experience in maternal and well-baby clinics or in postnatal wards. All eligible mothers and health care workers were verbally invited and written informed consent was obtained thereafter.

### 2.2. Data Collection

Six FGDs were carried out until new information was no longer generated; that is, data saturation was achieved [24] with each session lasting for 30–60 minutes [25]. Six IDIs were conducted; that is, one for each health care worker, each lasting approximately one hour. Two separate semistructured guides prepared by the research team were used to facilitate the FGDs and IDIs. The guides were developed by using the findings of a literature review, expert advice of a nutritionist, and information obtained through informal discussions carried out with 10 GDM mothers regarding dietary practices. The discussion guides were rephrased and finalized after pilot testing them on 5 mothers and 2 healthcare workers. The discussions and interviews were conducted in comfortable rooms in healthcare institutions in close proximity to the location of the participants. The same moderator, a postgraduate student (TS), facilitated all sessions, while the notetaker recorded all nonverbal and verbal expressions. All sessions were audiorecorded and transcribed verbatim.

### 2.3. Data Analysis

The framework approach [26] was used for data analysis. The entire research team checked all transcripts for errors by reading them and listening to the audiorecordings simultaneously. After familiarizing further with the transcripts and audiorecorded interviews, the coders (TS and CW) discussed the common and uncommon code categories with the team members. The coders independently coded the data of the 12 transcripts (6 of FGDs and 6 of IDIs) to minimize potential bias. Coding was compared between coders and differences were reviewed and resolved. The process of refining and applying was repeated until no new codes were generated. Once all data had been coded using the analytical framework, the data was abstracted from transcripts and summarized verbatim in Microsoft Excel in a matrix comprised of one row per participant and one column per code. Finally, the matrix was reviewed intensely and themes were generated.

## 3. Results

### 3.1. Participant Characteristics

A total of 30 postpartum mothers participated in focus group discussions and 6 healthcare workers (3 postnatal nurses and 3 field midwives) were interviewed. All mothers were either in their second or third pregnancy and had a past history of GDM. Twenty-four mothers (80%) were below 35 years. Sixty-seven percent of the samples have studied up to ordinary level while only 23% and 10% have studied up to advanced level and diploma/degree level, respectively. All midwives and nurses who participated in the study had a work experience of more than ten years. All mothers and health care workers reported that they felt comfortable and relaxed during the procedure.

### 3.2. Emerging Themes

Seven major themes emerged from focus group discussions and in-depth interviews.

#### 3.2.1. Theme 1: Myths and Traditions Specific to the Postpartum Period

In general, almost all mothers reported negative perceptions on consuming a variety of healthy food. Mothers emphasized the importance of consuming traditional food to maintain their health in the postpartum period. Inclusion or restriction of certain food items in the diet of the mother was influenced by traditional beliefs on top of scientific evidence.

All postpartum mothers believed that traditional foods, especially “thambunhodi” (a Sri Lankan soup made out of water and spices), are an excellent alternative to take with rice after delivery in spite of having curries. In the words of a mother regarding traditional foods,

*My mother, mother-in-law, and grandmother asked me to drink thambunhodi every day because it gives lot of energy as it is prepared using garlic, karapincha (curry leaves), and pepper.*

*We have prepared a special energetic drink which contains “Arrack” (an alcoholic beverage) and garlic. I drank it. It gave me additional energy and also helped to stop my vaginal bleeding.*

Restriction of certain food items during the postpartum period was another common practice which is related to the traditional beliefs. Mothers avoided eggs, small bony fish, and green leaves expecting an array of benefits during the postpartum period.

In the words of a mother,

*They did not allow me to eat small bony fish and green leaves as the bones can get stuck in my mammary glands obstructing the milk secretion.*

*My mother told me not to eat eggs as it increases the tendency for placental parts to remain in the womb. She also told to avoid fried meat and fish as if I eat them, demons would follow me.*

Health care workers reaffirmed the mothers' views on perceived health benefits of the chosen foods. One midwife stated,

*Actually, the mothers believe that “thambunhodi” is the best curry to reduce maternal complications because garlic and pepper have the magical ability to clean the gastrointestinal tract and improve immunity. Traditional alcoholic drink is the best energy source for mothers.*

Health care workers also commented that although they have educated the mothers regarding nutritious food, women usually consumed traditional food. One midwife stated,

*However much we educate them about healthy food, it's not an easy task to change their traditional views. To tell you the truth, actually we also drank “thambunhodi” during our postpartum period.*

*Although we advise them to take more proteins and iron-containing food, they like to eat food rich in starch like “kirikos” (mature jack fruit curry) and “polos” (tender jack fruit curry) without a limit as they believe that these foods increase production of milk. Mothers do not consider their food as important after the baby is born.*

The healthcare workers indicated that myths regarding food are related to religious beliefs of the community and the social influence on the mothers. One postnatal nurse stated,

*The mother, other relatives and neighbours always interfere with their dietary practices. People around them offer food to these mothers and to maintain the goodwill, most mothers consume what is offered.*

#### 3.2.2. Theme 2: Lack of Motivation

Mothers did not feel the necessity to restrict carbohydrates in the diet after the baby is born. Almost all mothers attributed restricting carbohydrates to reduced production of breast milk, lack of energy, and gastritis. They were not prepared to accept that they are vulnerable to developing T2DM in their later life. Several mothers stated this during the focus group discussions.

*My baby is no longer in my womb; therefore, why should I go for dietary modifications?*

*After the baby came out, my sugar level became normal. Therefore, there is no harm to me or my baby.*

*After my first pregnancy, nobody asked me to restrict carbohydrates and none of my friends did so. They did not develop any illness. So why should I do it?*

#### 3.2.3. Theme 3: Time Pressure

Time is a limited resource after the baby is born. Mothers mentioned that they do not have time to prepare separate food for themselves. It was observed that they eat anything available at home while attending to the needs of the baby. One mother said,

*I have a lot of work at home. I have to fulfil the demands of my baby and my husband. Every time my baby cries, I have to lull him and feed him. My husband also expects me to fulfil some of his duties. Nobody helps me. Hence, I have no time to prepare special food for me. Whole day I work like a machine. Twenty-four hours is not enough for me. I am so tired*.

#### 3.2.4. Theme 4: Financial Barriers

Another concern expressed by mothers was the financial difficulties. Twenty-eight mothers out of thirty expressed about their financial problems. As mothers stated, only one person earns in most families. Therefore, they have to choose food which is easily available like jack fruit and manioc (cassava). They consume these high-carbohydrate foods to achieve satiety as these foods were perceived as inexpensive and thus economically viable.

One mother stated,

*It is not easy to spend additional money for special food. I prepare common food for all family members. Some foods are very expensive. One hand to earn and many mouths to eat! How can I be selfish to prepare special food for me? It is not practical*.

Another mother noted,

*My mother-in-law and the unmarried sisters-in-law also depend on my husband's salary. Hence it is difficult to eat food items like fish or chicken every day. I prepare cheap food that can be obtained from our garden for all three meals.*

The healthcare workers confirmed that the families give priority in fulfilling the needs of the babies than the mothers. One postnatal nurse stated,

*Only one person earns for the family and this money is not enough to fulfil the family needs. Therefore, they spend money to fulfil baby's needs. Sometimes it is useless giving dietary advice to mothers because they are really poor. They eat cheap, easily available food items preparing one item for the entire family.*

#### 3.2.5. Theme 5: Negligence of Mothers and Families

Mothers indicated that they do not have an interest to follow healthy dietary modifications after the baby is born. The baby is the treasure and the focus of the family. Therefore, they are not concerned about the well-being of the mother. According to their point of view, diet does not have any effect after the baby is born.

*I have no interest to do it. What is the use of changing my diet? After the baby is born, why should I spend time thinking about my food? Although I have been advised to modify my diet, I am not interested and do not care about it.*

Healthcare workers endorsed the statement made by the mothers regarding healthy food practices. They indicated that after the birth of a baby, most mothers and family members do not care about the well-being of the mother. One public health midwife said,

*They think that there is no need to change their food habits because the baby is not in the womb any more. However much we try to educate them about healthy food practices, they don't care. When we give them instructions, they nod and seem to agree but when we inquire later whether they practiced, they just smile. Although they neglect themselves, they are willing to practice whatever advices given regarding the newborn*.

#### 3.2.6. Theme 6: Lack of Awareness Regarding GDM and Its Postpartum Dietary Recommendations

The healthcare workers indicated that not only the mothers but the healthcare workers themselves have limited knowledge about GDM and are not aware about the importance of postpartum dietary modifications for GDM mothers. In Sri Lankan society, the main focus is given to the antepartum period. As several health care workers explained,

*Actually, I am not aware about the relationship between GDM and DM. According to my knowledge, GDM is a disease which appears only during pregnancy and disappears when the baby is born. Therefore, there is no need to worry about GDM in the postpartum period.*

*To tell you the truth, we also do not have much knowledge about how a GDM mother should be managed in the postpartum period. We are not aware of the government health policy nor of recommendations for proper postpartum follow-up of these mothers. However, as a routine we advise the mothers to get a fasting blood sugar done at six weeks of their delivery.*

#### 3.2.7. Theme 7: Cultural Barriers

This theme has emerged mainly from healthcare workers. They explained that some Sri Lankan cultural beliefs are major barriers for a healthy diet.

One postnatal nurse stated,

*Our Sinhala culture promotes mothers to move to the maternal home soon after the baby is born. Then, the mature ladies, that is, the grandmother and mother decide what the new mother eats.*

One midwife stated,

*Our culture gives more priority to the new member who they think will protect their family name. The mothers' health is not so important after the baby is born.*

## 4. Discussion

From our semistructured focus group discussions and in-depth interviews, we identified seven main themes and recognized many factors which act as obstacles for women to practice healthy food habits. Discussions with mothers revealed several themes such as myths and traditions specific to the postpartum period, lack of motivation, time pressure, financial barriers, and negligence of mothers and families that hinder healthy food habits during the postpartum period. Lack of awareness regarding GDM and postpartum dietary recommendations for GDM mothers and cultural barriers surfaced solely from in-depth interviews with healthcare workers.

Careful exploration of the themes shows that as a result of many factors, almost all women end up consuming large portions of high carbohydrates while restricting foods rich in proteins, micronutrients, and fibre such as eggs, small fish, and green leaves. This not only compromises the nutritional needs of the mother who will be possibly lactating but also speed up the trajectory towards development of T2DM. In general, Sri Lankans are shown to consume high levels of carbohydrates and fats and low levels of protein and dietary fibres, which may have detrimental effects on health [27]. Therefore, it is likely that the myths, traditions, and practices even in the postpartum period are aligned with the general trend of food habits in the population. Focus group findings suggest that the myths regarding postpartum diet are perpetuated through generations possibly supported by the health care workers [22]. In addition, almost all women in the present study identified the mismatch between their income and food prices as a main inhibitor for healthy food choices. A previous qualitative study done among mothers with a history of GDM in Atlanta has also reported financial barriers as a major drawback for sustaining healthy eating behaviours [18]. This will further aggravate the situation as carbohydrate-rich foods such as jack fruit that is often consumed by these mothers are easily available even in their home gardens or can be purchased at low cost.

Mothers have identified lack of motivation and negligence as major barriers for healthy eating. Both self-motivation and social motivation appear to be poor among women who participated in focus group discussions. Similarly, studies conducted elsewhere have also shown that the mothers lose the motivation and interest for healthy behaviour after the delivery of the baby [16, 18]. In the present study, “Lack of awareness” has emerged during in-depth interviews with healthcare workers as a factor affecting dietary behaviour in the postpartum period. Similar to our findings, studies conducted elsewhere also reported that most GDM mothers were unaware of their risk of developing T2DM [18, 19] which can be considered as a barrier for lifestyle modifications. Lack of awareness regarding the link between GDM and T2DM may be considered as the root cause for the lack of motivation and negligence.

In the present study, health care workers stated that lack of clear guidelines was one of the main barriers in achieving lifestyle changes among postpartum mothers. Lack of awareness of health care workers seen in this study as well as in studies done in other settings [19] is a major drawback in postpartum management of these mothers. In many settings, women with GDM were found to receive close medical monitoring only during the pregnancy with inadequate postpartum follow-up and support from health professionals [20, 21, 28]. Our study also revealed that mothers get minimum inputs from health professionals during the postpartum period regarding healthy food practices, a finding confirmed even by researchers elsewhere [28, 29]. This suboptimal follow-up has the potential to downplay the significance of GDM as a risk factor for developing T2DM in the future.

The postpartum period is unique globally as these mothers are faced with childcare demands which are tiring [16]. As in other studies [19, 30], the lack of time was also identified by our mothers as a barrier to comply with healthy food choices. Therefore, all these factors should be considered when designing behaviour change strategies to suit this special group of women [14, 17]. Diet modification programmes should be culturally acceptable and aimed at all literacy levels [13] and delivered and followed up in innovative ways. Previous research has suggested home-based programmes [14] delivered and monitored through telephone calls [17, 23] due to time constraints and difficulties in transportation. Since the lack of social support has been identified as a barrier to implement a healthy diet [18], a collective role of all health care professionals catering to these women [19], their partners [30], and other family members [31] is of utmost importance. Although health care workers in the present study reported a lack of proper postpartum dietary guidelines for GDM mothers, a Canadian study reported that even in the presence of guidelines, there is a mismatch between the recommendations and the diet consumed by them [30]. This shows that adherence to guidelines needs continuous support and monitoring. Although the period of pregnancy could be used as an opportunity to increase the awareness of mothers regarding the future risk of DM [20], the support should continue in the postpartum period. Although women in some settings desired to learn strategies to attenuate future diabetes [32], additional efforts by healthcare personnel are needed if mothers do not show interest as in the present study.

Inclusion of two stakeholder groups from the same setting is a strength which enabled pooling of ideas to get a better understanding of the situation. In a multicultural country, inclusion of only one cultural group is considered a limitation as ideas of ethnic minorities would have explored different perceptions and barriers. However, the authors felt that the possibility of not disclosing information during focus group discussions was minimized as these mothers had the same experiences. The study had to be limited to only three districts in the country due to logistic reasons.

## 5. Conclusions

The findings have paved the way to understand the existing knowledge, common practices, and attitudes of society about dietary behaviours of postpartum mothers with a history of GDM. The information that emerged will be useful in developing culturally acceptable and scientifically valid dietary protocols for postpartum mothers with GDM to attenuate its progression to T2DM. Addressing the concerns of mothers is expected to increase acceptance and long-term adherence.

Lack of optimum awareness of not only mothers, but healthcare workers seen in our study as well as in other studies [19], seems to be a serious issue which needs immediate remedial action. Dissemination of valid information to mothers by health care workers should be ensured by appropriate awareness programmes. Close monitoring of GDM mothers during the postpartum period is a responsibility of all stakeholders. The barriers unique to postpartum mothers have to be considered when planning and monitoring follow-up.

## Data Availability

The Qualitative data used to support the findings of this study are available from the corresponding author upon request.
